# Hemispheric Differences in Corticospinal Excitability and in Transcallosal Inhibition in Relation to Degree of Handedness

**DOI:** 10.1371/journal.pone.0070286

**Published:** 2013-07-25

**Authors:** Travis Davidson, François Tremblay

**Affiliations:** 1 School of Human Kinetics, University of Ottawa, Ottawa, Ontario, Canada; 2 School of Rehabilitation Sciences, University of Ottawa, Ottawa, Ontario, Canada; 3 Bruyère Research Institute, Ottawa, Ontario, Canada; University of California, Merced, United States of America

## Abstract

In this study, we examined hemispheric differences in corticospinal excitability and in transcallosal inhibition in a selected group of young adults (n = 34) grouped into three handedness categories (RH: strongly right-handed, n = 17; LH: strongly left-handed, n = 10; MH: mixed-handed, n = 7) based on laterality quotients (LQ) derived from the Edinburgh Handedness Inventory. Performance measures were also used to derive a laterality index reflecting right-left asymmetries in manual dexterity (Dext_li_) and in finger tapping speed (Speed_li_). Corticospinal excitability was assessed in each hemisphere by means of transcranial magnetic stimulation (TMS) using the first dorsal interosseus as the target muscle. TMS measures consisted of resting motor threshold (rMT), motor evoked potential (MEP) recruitment curve (RC) and the contralateral silent period (cSP) with the accompanying MEP facilitation. Hemispheric interactions were assessed by means of the ipsilateral silent period (iSP) to determine the onset latency and the duration of transcallosal inhibition (i.e., LTI and DTI). Analysis of hemispheric variations in measures of corticospinal excitability revealed no major asymmetries in relation to degrees of laterality or handedness, with the exception of a rightward increase in rMTs in the LH group. Similarly, no clear asymmetries were found when looking at hemispheric variations in measures of transcallosal inhibition. However, a large group effect was detected for LTI measures, which were found to be significantly shorter in the MH group than in either the LH or RH group. MH participants also tended to show longer DTI than the other participants. Further inspection of overall variations in LTI and DTI measures as a function of LQs revealed that both variables followed a non-linear relationship, which was best described by a 2^nd^ order polynomial function. Overall, these findings provide converging evidence for a link between mixed-handedness and more efficient interhemispheric communication when compared to either right- or left-handedness.

## Introduction

The concept of handedness comes from the observation that most humans exhibit a preference for one hand over the other, with ∼90% of the population showing a rightward preference [1]. The question thus arises as to whether this asymmetrical hand use is reflected at the level of sensori-motor organization and in terms of neural control. Given the critical role of the primary motor cortex (M1) and its corticospinal projections in controlling fine aspects of manual dexterity, one would assume that asymmetries would also be reflected at this level. Yet, such a simplistic assumption has proven difficult to establish. At the anatomical level, structural imaging studies have produced rather inconsistent results with regard to asymmetries related to handedness, although many studies point to a specific leftward asymmetry in the size of the M1 region in consistent right-handers, particularly in male subjects [2]–[4]. The left M1 region also displays more profuse intrinsic connectivity in consistent right-handers [5]. These anatomical findings did not, however, translate into corresponding asymmetries at the physiological level. For instance, only minor asymmetries were found when comparing sensorimotor activation levels elicited during movement of either the preferred hand or the less preferred hand [6], [7]. When asymmetries are found they often go in the direction of a greater activation in the non dominant hemisphere [see 6 for a review]; a finding that could be explained by the fact that movements of the less preferred hand are more demanding and thus require greater control.

Transcranial magnetic stimulation (TMS) studies of hemispheric differences in cortical excitability have also produced rather conflicting results with regard to handedness. For example, while some reports found asymmetries in motor threshold (MT) with lower values for the dominant hemisphere [8], others reports did not find such asymmetries [9], [10]. In the same vein, some reports pointed to an asymmetry in the strength of interhemispheric inhibition from the dominant over the non-dominant M1 in right-handers [11] but this finding could not be confirmed by others [12]. On the other hand, TMS studies of task-dependent changes in corticospinal excitability have provided more consistent results in line with those from neuroimaging studies in showing greater corticomotor facilitation in the non dominant M1 when the less preferred hand was involved [13], [14]. The overall picture that emerges from the TMS literature on handedness is one of difficulty establishing a strong link between the observed behavioural asymmetry in hand use and its neurophysiological correlates at the corticomotor level. As pointed out by Bernard et al. [15], beyond variations in experimental protocols, one major reason for the conflicting evidence is the fact that most authors have considered handedness only in terms of direction (i.e., either right or left preference) without consideration for the degree of lateralization (i.e., how strong is your preference). Interestingly, the relatively few studies that have considered the degree of handedness have produced much more consistent findings. For instance, Dassonville et al. [16] used the Edinburgh Handedness Inventory [17] to quantify the degree of hand preference in both right- and left-handers and showed that the greater the degree of handedness the greater the lateralized difference in motor cortex activation during use of the dominant hand. More recently, Martin et al. [18] made similar observations in showing that the parieto-cortical network activated during grasping was largely asymmetrical and determined by the degree of lateralization in either strongly right-handed or strongly left handed individuals. In a TMS study, Triggs et al. [8] used right-left differences in dexterity tests to quantify the degree of lateralization in groups of right-handers and left-handers. This index of handedness provided the best correlation with corresponding measures of right-left asymmetry in MT (i.e., the larger the manual asymmetry, the greater the threshold asymmetry). Thus, one way to tackle issues related to handedness and its neurophysiological correlates is to move beyond a simple right-left dichotomy to examine the whole spectrum of handedness using preference and performance measures to assess how strongly one is handed.

In the present study, we sought to further investigate the neurophysiological correlates of handedness using a set of TMS measures to characterize not only hemispheric differences in basic measures of corticospinal excitability but also differences in interhemispheric inhibition. To examine the influence of handedness, we used hand preference and performance measures to characterize the degree of handedness in our participants. Given recent evidence pointing to a relationship between degree of laterality and asymmetries at the cortical level, we hypothesized that hemispheric differences would emerge in individuals exhibiting strong preference for one hand (either right or left) and large manual asymmetries in performance when compared to individuals with no clear preference for one hand (i.e., mixed handedness) and lower degrees of manual asymmetries.

## Materials and Methods

### Ethics Statement

The study procedures were approved by the Research Ethics Board at the Bruyère Research Institute, Ottawa, Ontario, Canada. Written informed consent was obtained prior to participation from all participants in accordance with the *Declaration of Helsinki*. All assessments were performed in a controlled laboratory environment. Each participant received a small honorarium for his or her participation.

### Participants

Thirty-four young healthy adults (18–30 years) were recruited for this study from the community in the Ottawa-Gatineau area. Participants were initially recruited on the basis of self-report of handedness. During the process, a special effort was made to recruit left-handed participants so that both hand preference groups would be adequately represented in our pool of participants. The final sample included 19 self-reported right-handers (8 females) and 15 self-reported left-handers (9 females). Before testing, all participants completed a medical questionnaire to assess their general health and to ensure that there were no contra-indications to TMS. The demographic characteristics of participants are described in Table 1.

**Table 1:** Characteristics of the participants with respect to hand preference and manual performance.

	Left Handed	Mixed handed	Right Handed
	(LQ≤−75)	(−75≤LQ≤+75)	(LQ^a^≥+75)
	(n = 10)	(n = 7)	(n = 17)
Age (years)	21.1±1.9	24.0±3.1	21.6±2.6
(range)	(18–25)	(22–30)	(19–29)
Gender	2 M, 8 F	5 M, 2 F	10 M, 7 F
Dexterity			
GPT (s)	RH: 60.0±6.3	RH: 56.6±4.4	RH: 54.6±6.3
	LH: 52.5±4.6	LH: 56.3±9.0	LH: 66.3±9.2
Dext_li_ b	6.6±5.8	0.7±6.4	−9.9± −4.4
Execution Speed			
FTT (#taps/15 s)	RH: 94.4±12.5	RH: 96.6±14.2	RH: 100.5±11.5
	LH: 98.7±9.5	LH: 101.3±14.9	LH: 90.4±8.3
Speed_li_ c	−2.4±4.0	−2.3±4.7	6.2±4.5

Values are given as mean and standard deviation.

a Laterality quotient (LQ =  (Right-Left)/(Right+Left)×100) derived from self-report of hand preference with the Edinburgh Hand Inventory.

b Laterality index derived from performance in the Grooved Pegboard Test (GPT)) reflecting right-left asymmetries in dexterity (Dext_li_ = Right-Left)/(Right+Left )×100).

c Laterality index derived from performance in the Finger Tapping Test (FTT) reflecting right-left asymmetries in speed of execution (Speed_li_ = (Right-Left)/(Right+Left )×100).

Abbreviations: M: Male; F: Female; RH, Right Hand; LH, Left Hand.

### Hand Preference Groups and Degrees of Handedness

To reflect differences in the degree of hand preference (HP), participants were divided into three HP groups on the basis of laterality quotients (LQs) computed from the Edinburgh Handedness Inventory as: (Right-Left)/(Right+Left)×100 [17]. We used the upper (LQ>+75) and lower quartiles (LQ<−75) respectively, to assign participants to either a strong right-handed group (RH, n = 17) or a strong left-handed group (LH, n = 10). The remaining were assigned to a mixed-handed group (MH, n = 7). Besides LQs derived from the Edinburgh Inventory, we computed two others laterality indices from measures of manual performance. The first index reflected right-left asymmetries in dexterity as measured with the Grooved Pegboard Test (GPT, Lafayette Instrument Co, IN 47903). The GPT consists of inserting 25 small pegs into keyhole-like grooves as fast as possible using a fine precision grip. Each hand was tested once and the timed performance in seconds (s) to complete the test (i.e. 25 peg insertions) was used to derive a dexterity laterality index (Dext_li)_ computed as: (Right_GPT_-Left_GPT_)/(Right_GPT_+Left_GPT_)×100. Note that since the GPT reflects a timed performance, a positive Dext_li_ corresponds to better performance with the left hand whereas a negative Dext_li_ corresponds to better performance with the right hand. The second index assessed right-left asymmetries in the speed of execution with the Finger Tapping Test (FTT). The FTT was administered using a MoART panel with the accompanying PsymSoft II™ software (Lafayette Instruments Co., IN 47903). The task consisted of tapping a circular target at the center of the board successively for 15 seconds with the index finger as rapidly as possible. Participants were instructed to focus on speed and not on accuracy. As for the GPT, each hand was tested once (order counterbalanced) and the performance in terms of number of valid taps (i.e., hitting the target) was used to derive a speed laterality index (Speed_li)_, which was calculated as: (Right_FFT_-Left_FFT_)_/(_Right_FFT_+Left_FFT_)×100.

### Transcranial Magnetic Stimulation (TMS) and Motor Evoked Potentials (MEPs)

TMS was administered with participants comfortably seated in a recording chair. Magnetic stimulation was delivered with a Rapid^2^ stimulator (Magstim Co. Dyfed, UK) connected to a figure-eight coil (90 mm outer loop diameter). MEPs were recorded using small auto-adhesive surface electrodes (Ag/AgCl, Kendall Medi-Trace™ 130) placed over the first dorsal interosseous (FDI) muscles of the right and left hand. Electromyographic signals were amplified and filtered with a time constant of 10 ms and a low-pass filter of 1 kHz (AB-621G Bioelectric amplifier, Nihon-Kohden Corp., CA 92610). Signals were digitized at rate of 2 kHz (BNC-2090, National Instrument Corp.) and further relayed to a laboratory computer running custom software to control acquisition.

To determine the optimal site to evoke MEPs in the contralateral hand muscles, participants were fitted with a Waveguard TMS compatible cap (ANT North America Inc, WI 53719). A U-shaped neck cushion was also used to restrain head movements and prevent neck fatigue. With the coil held ∼45° in the mid-sagittal plane, the approximate location of the hand motor area on the tested hemisphere was explored in 1-cm steps until reliable MEPs could be evoked in the target muscle. This site was then marked with a sticker to ensure consistent coil positioning. After determination of this stimulation “hotspot”, the coil was held in place manually by one of the experimenters (FT) to derive specific measures of corticospinal excitability. The experimenter frequently reassessed the coil position to ensure that it remained over the optimal stimulation site throughout the experiment. All TMS testing sessions took place between 9am and 4pm to avoid diurnal variations in corticospinal excitability [19].

### Measures of Corticospinal Excitability

In each participant, specific measures of corticospinal excitability were derived from each hemisphere, the order of testing between the two alternating between participants. The first measure consisted of the resting motor threshold (rMT), which reflects neuronal membrane excitability in a given motor representation [20]. For rMT determination, we used the procedure described by Mills and Nithi [21] which consisted of determining a upper (10/10 MEPs) and a lower threshold intensity (0/10 MEPs) following a series of single TMS pulses and taking the median as the rMT intensity. Such a method has been shown to lead to more reliable and accurate estimates of rMT than the conventional method of using the intensity at which 50% MEPs are elicited [22]. The second measure of excitability was the recruitment curve (RC) at rest, which describes the relationship between MEP amplitude and TMS intensity. The RC reflects the strength of corticospinal projections and the extent of a given motor representation [20]. Single TMS pulses at 90%, 100%, 110%, 120% and 130% of rMT were applied consecutively and 5–10 MEPs were recorded at each stimulation intensity. For both rMT and RC procedures, EMG activity was constantly monitored on a high gain oscilloscope to make sure that unwanted contractions did not interfere with the measurements. The third measure was obtained during active contraction and consisted in the contralateral silent period (cSP) with the associated MEP facilitation (MEP_facil_). For the cSP, single TMS pulses at 120% rMT were delivered while participants exerted a constant static force (duration 5 s) at 25% of their maximal strength with a pinch gauge. Five trials were performed for each hand/hemisphere.

### Measures of Transcallosal Inhibition

To examine hemispheric interactions, we used the ipsilateral silent period (iSP), to assess transcallosally-mediated inhibition between motor cortices [23]. To elicit the iSP, we used the approach described by Giovannelli et al. [24], whereby single TMS pulses (120% rMT) were delivered ipsilaterally to the maximally contracting hand (maximal force exerted on the pinch gauge), while the opposite hand exerted a light force by gently squeezing a soft ball between the thumb and index fingers (∼15% of the maximal activation). This procedure was repeated five times for each hand/hemisphere.

### TMS Data Analysis

TMS data were analysed off-line by the same investigator (TD) using numerically coded files to avoid any biasing with regard to HP groups. To assess RCs, MEPs evoked at each intensity were measured peak-to-peak to obtain mean MEP amplitudes. Then, the mean amplitude was plotted against TMS intensities to obtain the RC. As suggested by Ray et al. [25] we used linear regression analyses to characterize the relationship between MEP amplitude and TMS intensity. To improve the goodness of fit and to account for large inter-individual variations, we used squared root transformation of MEP values to assess the RC. Such transformation greatly improved the goodness of fit of the relationship (averaged *r^2^*: untransformed, 0.89±0.6; transformed, 0.94±0.05*).* For statistical comparisons, we used the slope of the RC as a simple summary statistic for the RC relationship at rest [25]. For the cSP, we performed a trial-by-trial analysis to estimate its duration and to measure the amplitude of the associated MEP (MEP_facil_). The duration of the cSP was determined in each trial in line with guidelines from previous studies [e.g., see 26,27] as the time interval from the onset of the MEP to the return of at least 50% of the mean pre-stimulus background EMG activity. From this analysis, mean values were computed for the cSP duration and the MEP_facil_ amplitude by averaging all trials for each hand/hemisphere. As for MEPs recorded at rest for the RC, the amplitude of facilitated MEP_s_ varied greatly between individuals (skewness>3.0, Shapiro-Wilk’s normality test, p<0.01) and thus were subject to log-transformation to normalize the distribution [28]. For iSP recordings, we adopted the same trial-by-trial analysis as for the cSP to derive two specific measures of transcallosal inhibition. First, the iSP onset, which reflects the onset latency of transcallosal inhibition (LTI), was determined as the time from the stimulus onset until the 1^st^ sign of significant decline (>25%) in the mean rectified EMG activity level. The second index come from determining the iSP duration by measuring the time in ms from the iSP onset until the 1^st^ sign of recovery in the background EMG activity (i.e., iSP offset). The latter time point is relatively easy to determine, as the end of the myoelectric silence is generally followed by an abrupt return of EMG activity in the recovery period (see RESULTS, for examples of iSP recordings).

### Statistical Analysis

To determine how handedness categories influence measures of intra-hemispheric excitability and interhemispheric inhibition and how these measures co-vary with laterality indices, we performed a series of repeated measures Analysis of Covariance (ANCOVA) using “hand/hemisphere” (right vs. left) as the repeated factor, HP group (RH, LH, MH) as the between-subjects factors and the two laterality indices (Dext_li_, Speed_li_) as co-variates. The significance level was set at p<0.05 for detection of main effects and interactions. Post-hoc comparisons were performed using Tukey’s test. Planned comparisons were also performed to examine specific combinations using t-tests (paired and unpaired, Bonferroni-adjusted to reduce Type I errors). Linear regression analyses were used to examine the relationships between laterality scores derived from preference and performance measures. Most analyses were performed using SPSS software version 17.0 for Windows^®^ (Chicago, IL, USA). GraphPad Prism version 5.00 for Windows (GraphPad Software, San Diego California USA, www.graphpad.com) was used to prepare illustrations and to perform secondary analyses dealing with non-linear curve fitting.

## Results

### Manual Performance and Laterality Index

**Figure 1:**
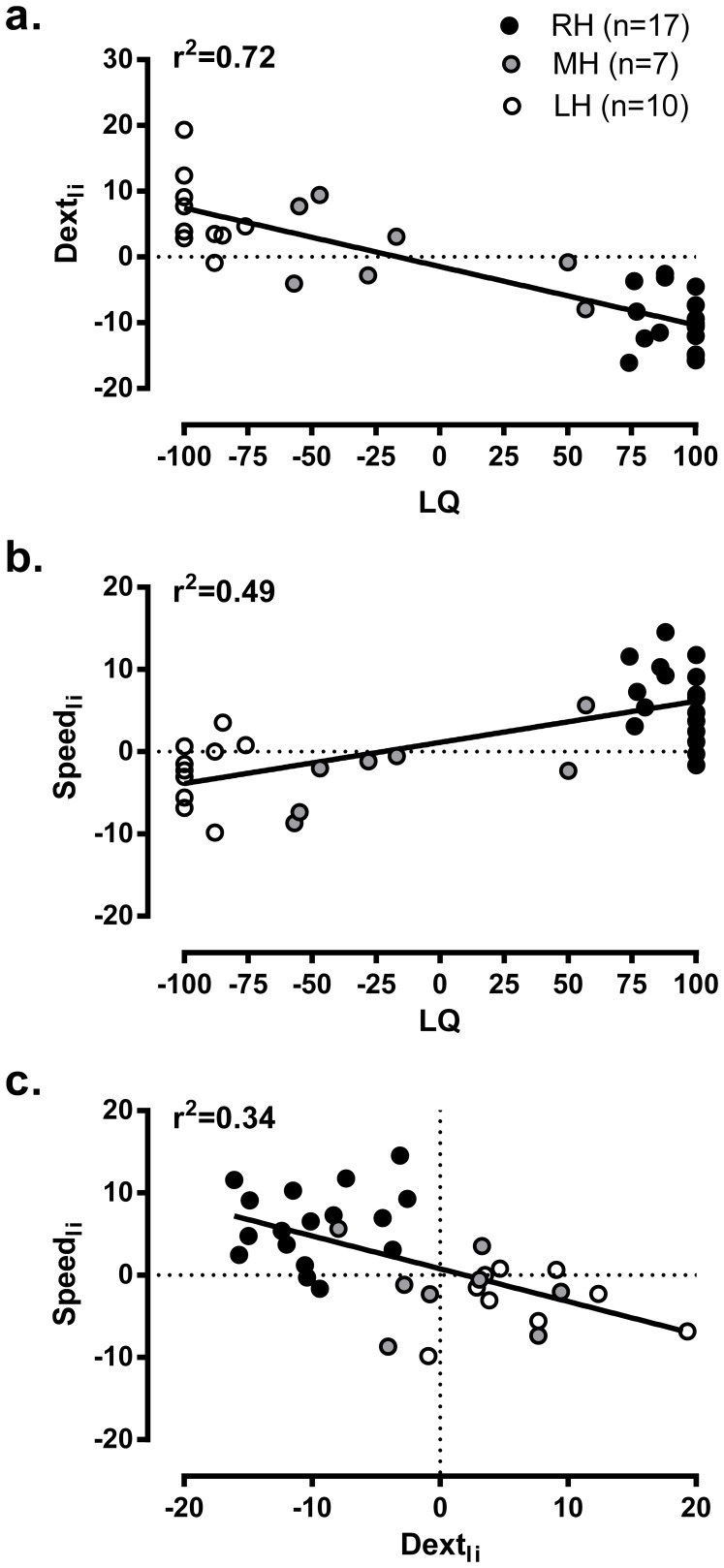
Relationships between preference and performance measures. a and b. Association between laterality quotients (LQ) computed from the Edinburgh Handedness Inventory and laterality indices computed from performance in the dexterity (Dext_li_) and finger tapping tests (Speed_li_), respectively. **c.** Association between the two laterality indices derived from performance tests is shown in c. All laterality indices were computed as: (Right-Left)/(Right+Left)×100.

The performance in the behavioural tests to assess laterality, along with corresponding indices, is shown in Table 1 for the three HP groups. As expected, both RH and LH groups exhibited strong asymmetries on the two tests, which were reflected in the behavioural laterality indices. In contrast, the MH group showed correspondingly less asymmetry, particularly in the dexterity test. The relationship between participants’ perceived degree of handedness, as indexed by LQs, and indices of laterality measured from behavioural tests can be further appreciated in Figure 1. As evident in the Figure (a and b), the strength of the association between LQs and actual asymmetries in manual performance was greater for the dexterity test than for the FTT. Indeed, LQs accounted for >70% of the variance in Dext_li_, whereas they accounted for >50% of the variance in Speed_li_. As for the relationship between the two behavioural indices (Figure 1c), they showed only a moderate degree of association, indicating that the two were somewhat divergent in reflecting manual asymmetries associated with handedness.

### Hemispheric Differences in Corticospinal Excitability

**Figure 2:**
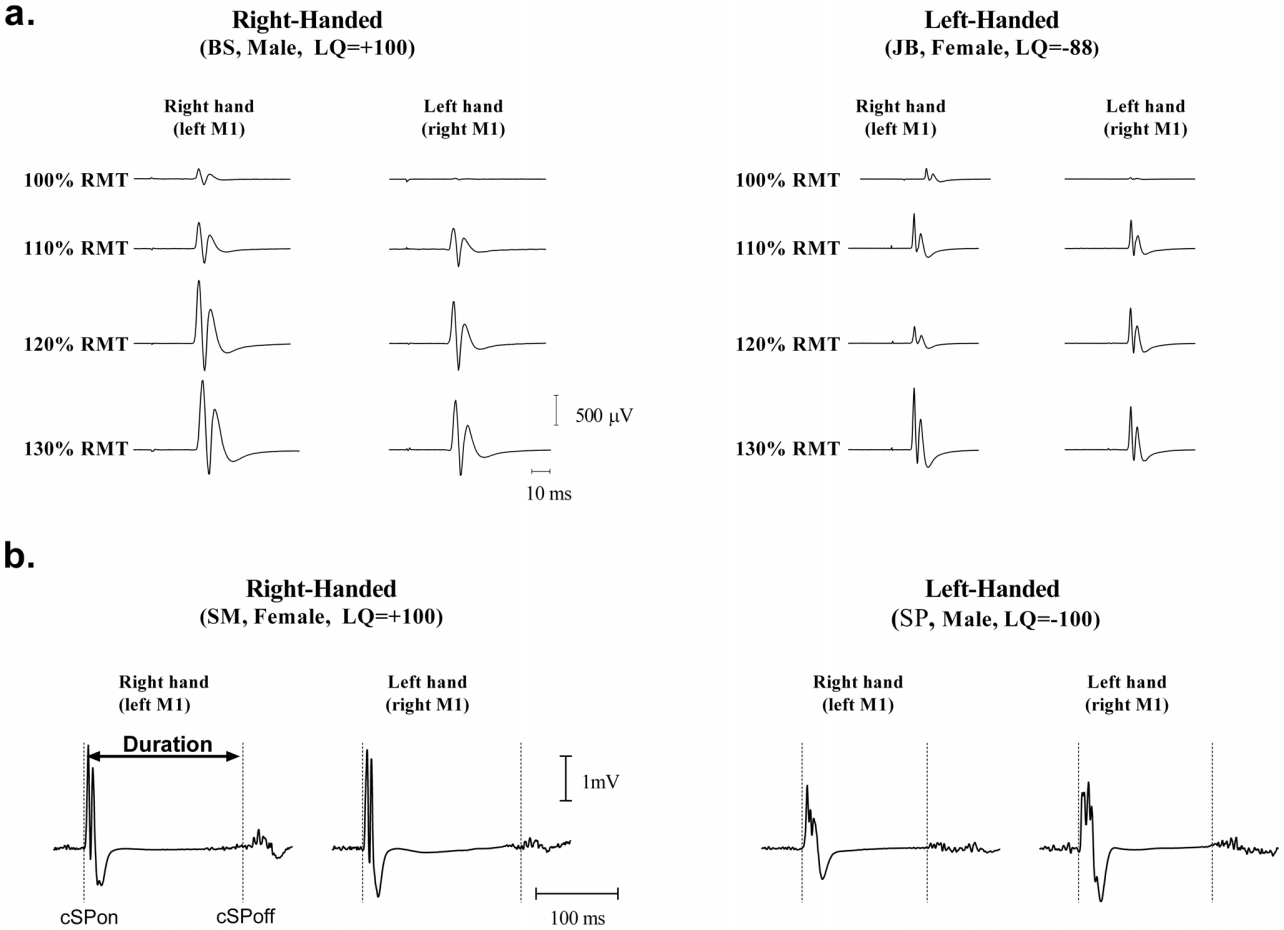
Hemispheric differences in corticospinal excitability in right-handed and left-handed participants. **a.** Examples of motor evoked potentials (MEP) amplitude recruitment in response to increasing stimulation intensity. **b**. Examples of recordings of contralateral silent period (cSP) obtained during active contraction with associated MEP facilitation. The two vertical dotted lines illustrate the approximate time for the onset and offset of the cSP. Note the relative symmetry in neurophysiological responses between the two hemispheres (**a** and **b)**. Abbreviations: RMT, resting motor threshold, LQ: laterality quotient.

**Figure 3:**
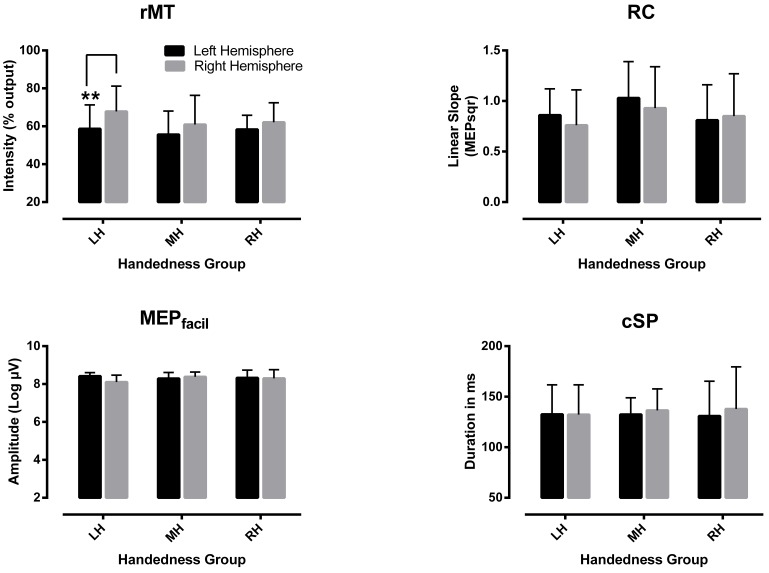
Mean variations (±1 SD) in measures of corticospinal excitability recorded between hemispheres in each group. In each graph, columns represent mean values computed from each hemisphere for all participants within each hand preference group (LH, left-handed group (n = 10), MH, mixed-handed group (n = 7); RH, right-handed group (n = 17). Abbreviations: rMT, resting motor threshold; MEP_facil_, motor evoked potential facilitation during active contraction, RC, recruitment curve; cSP, contralateral silent period.** significant right-left difference as detected with a paired t-test.

In general, only small differences were observed between hemispheres in measures of corticospinal excitability, irrespective of HP. Typical examples of right-left variations in MEP amplitude at increasing TMS intensities are shown in Figure 2 (a) along with examples of cSP recordings (b). It can be seen that for both the RH and LH participants, the variations recorded were largely comparable between hemispheres. In fact, the only noticeable asymmetry found was in rMT, which tended to be higher on the right as compared to the left hemisphere in all participants. This threshold asymmetry is evident in Figure 3, where averaged variations in TMS measures computed for each hand/hemisphere are shown for each group. The ANCOVA confirmed the presence of a large effect of “hand/hemisphere” on rMT (F_1, 29_ = 30.0, p<0.001), although no other interaction or main effects were detected (F_1, 29_<0.5, p>0.49). Planned comparisons revealed a significant right-left difference (*t*_9_ = 6.8, p<0.001) in rMT only in the LH group; the other two HP groups showing only trends for significance at the adjusted p-value (i.e., p = 0.016; RH group, t = 2.1, p = 0.052; MH group, t = 2.7, p = 0.04). Besides this threshold asymmetry, no other main effect or interactions were detected for the other remaining TMS measures (RC, F_1, 29_<1.35, p>0.26; MEP_facil_, F_1,29_<2.5, p>0.11; cSP, F_1, 29_<0.69, p>0.40) (Figure 3).

### Hemispheric differences in Transcallosal Inhibition

**Figure 4:**
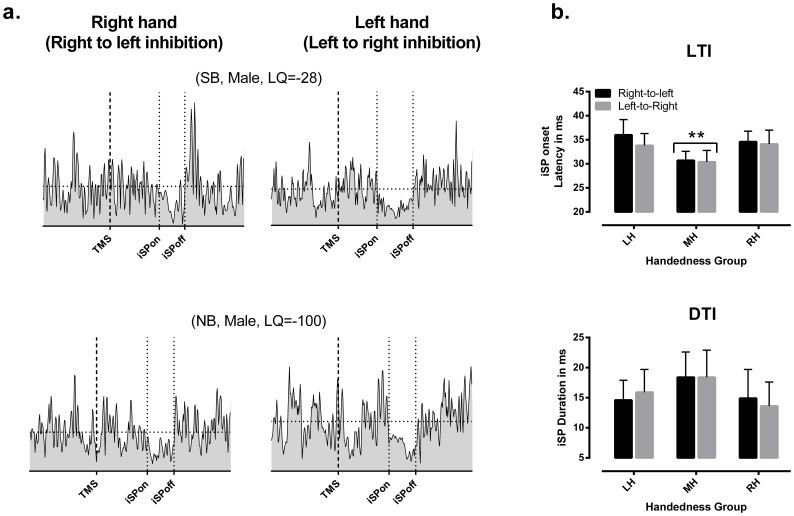
Hemispheric differences in transcallosal inhibition. **a.** Examples of ipsilateral silent period recordings (iSP) from a mixed-handed (MH) participant and a left-handed (LH) participants. In each recording, the averaged rectified electromyographic activity is shown to illustrate the approximate time points when the period ipsilateral inhibition was induced (i.e., iSP onset) and for how long it was maintained (i.e., iSP duration) after the stimulus delivery (1^st^ thick dotted lines). The time delay between the 1^st^ and 2^nd^ dotted lines corresponds to the latency onset of transcallosally-mediated inhibition (LTI), whereas the time period delimited by 2^nd^ and 3^rd^ dotted lines corresponds to its duration (DTI). Note the earlier onset and the longer period of ipsilateral inhibition in the MH participant when compared to the LH participant. **b**. Mean variations (±1 SD) in measures of transcallosal inhibition recorded between hemispheres for each group. Note the significant difference (**p<0.01) observed between groups in LTI. A corresponding trend is also observed for longer DTI in the MH group, but this trend could not be confirmed statistically.

As for measures of corticospinal excitability, measures of transcallosal inhibition derived from iSP recordings (i.e., LTI and DTI) were found to be largely symmetrical between hemispheres in all participants. However, some interesting differences emerged between groups. Such differences are illustrated in Figure 4 (a), showing examples of iSP recordings from two participants, one from the MH group and one from the LH group. In the MH participant, it can be seen that the onset of the ipsilateral inhibition (i.e., LTI) tended to be earlier and the period longer that than recorded in the LH participant. This difference in LTI was confirmed in the ANCOVA, where a highly significant group effect (F_2,29_ = 10.3, p<0.001) was detected; this factor alone accounting for >40% of the total variance. As shown in Figure 4 (b), post-hoc comparisons confirmed that LTI measures from the MH group were significantly shorter (p<0.001) than those derived from either the RH or LH group. In line with this, DTI measurements also tended to be longer in the MH group (Figure 4a), but this trend could not be confirmed in the ANCOVA (F_2, 29_ = 1.61, p = 0.21).

**Figure 5:**
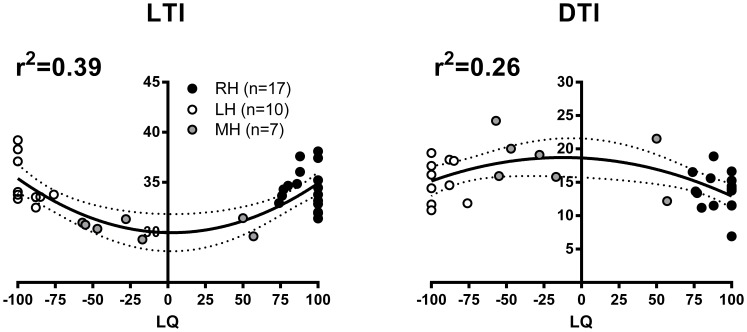
Variations in measures of transcallosal inhibition (onset latency and duration) as a function of laterality quotients (LQ) across all participants. Note the non-linear nature of the relationship for each variable, which fits with a 2^nd^ order polynomial function. Note the relatively good fit, as judged from *r^2^* values, for variations in LTI measures and, to a lesser extent, with DTI measures. The dotted lines represent the 95% confidence interval.

Further examination of overall variations in measures of transcallosal inhibition with respect to LQs revealed an interesting relationship. This relationship is illustrated in Figure 5, showing the distribution of individual LTI and DTI measures, after averaging right and left values, against corresponding LQs. It can be seen that participants with weaker degrees of handedness lateralization tended to show earlier LTI and longer DTI as compared to those with stronger degrees in either the rightward or leftward direction. Also evident in Figure 5 is the fact that each variable follows a non-linear distribution that matches to a large extent the distribution of LQs. In fact, curve-fitting analysis revealed a very good fit with a second order polynomial function for variations in LTI and a relatively good fit for variations in DTI (Figure 5). The same analysis performed with the two laterality indices revealed only a poor fit, however, for both LTI (Dext_li_, r^2^ = 0.01; Speed_li_, r^2^ = 0.03) and DTI (Dext_li_, r^2^ = 0.02; Speed_li_, r^2^ = 0.10).

## Discussion

In this study, we examined hemispheric differences in selected measures of corticospinal excitability and interhemispheric inhibition to examine the influence of handedness in a group of participants who displayed different degrees of laterality as assessed by preference and performance measures. It was hypothesized that asymmetries would be revealed in individuals exhibiting higher degrees of lateralization either for the right or left hand when compared to those exhibiting lower degrees of lateralization. In general, our results did not support this hypothesis and revealed no major hemispheric asymmetries in relation to the degree of handedness for basic measures of corticospinal excitability, with the exception of rMT. Similarly, measures of transcallosal inhibition were found to be largely comparable between hemispheres and showed no asymmetry in relation to handedness. However, the same measures exhibited a distinct pattern of variations when compared across HP groups. In the next section, we will first examined issues related to measures and classifications of handedness. Then, we will interpret the significance of the present findings for the study of the neurophysiological correlates of handedness.

### Measures of Handedness

In the present study, we attempted to address the issue of the neurophysiological correlates of handedness by moving from the simple right-left dichotomy to a three-way classification. However, one critical issue that arises when attempting to split handedness into more than two categories pertains to the definition of mixed handedness. In this study, we used the upper and lower quartiles of LQs as boundaries to sort out consistent handers (RH and LH groups) from less consistent handers (MH group). In this respect, our categorization is in line with the analysis performed by Dragovic [29] who demonstrated that a LQ of ±60 provided the best cut-off to separate mixed-handedness from right- and left handedness. In fact, in our MH group, LQs ranged from −55 to +57, which falls exactly within the optimal range described by Dragovic. According to the same author, such a cut-off point would allow extraction of ∼20% of mixed-handed individuals in a given population, which represents the exact proportion extracted in this study (i.e., 7/34, 21%). Finally, the frequent observation that mixed handedness is closely associated with left handedness [30] was also confirmed in our MH group since the majority (5/7) displayed a leftward preference. Thus, our MH group exhibited the expected characteristics of individuals who show no clear preference for one hand, as reported in other studies using similar handedness categorization [29]–[31]. The fact that our classification represented valid categories along the handedness continuum was further confirmed by the high degree of correspondence found between preference measures (LQs) and actual asymmetries in performance revealed in behavioral tests. In this regard, the Dext_li_ was clearly superior to the Speed_li_ in reflecting the association between reported preference and actual performance. Similar observations with regard to the superiority of dexterity over tapping speed to assess differences in laterality have been made in other report where such associations have been examined [e.g., 30,32]. To summarize, our approach to categorize handedness using both preference and performance measures allowed us to identify a small subgroup of mixed-handed participants displaying the expected characteristics of individuals who are typically less lateralized than the majority. Interestingly, it is in this subgroup where significant differences were found at the neurophysiological level.

### Handedness and Hemispheric Differences in Corticospinal Excitability

Contrasting with manual performance, basic measures of corticospinal excitability were largely comparable between hemispheres and were little influenced by the degree of handedness. In fact, the only asymmetry found was for a rightward elevation in rMTs in the LH group. In this regard our observations appear largely consistent with those of Bernard et al. [15], who performed similar comparisons of measures of corticomotor excitability (i.e., rMT, MEP size, motor mapping) and found no major asymmetries as a function of handedness using the same three-way classification as we used. As stated earlier, TMS studies have produced mainly conflicting evidence to support the existence of a strong link between hand dominance and laterality differences in corticospinal excitability [see 6 for a review]. For example, Livingston et al (2010) examined the influence of hand dominance, among other factors (e.g., gender), on interhemispheric differences using several TMS measures at rest, including rMT and MEP amplitude, in relatively large groups of right and left-handers and concluded that handedness had little influence. Bäumer et al. [33] reach a similar conclusion with regard to the influence of hand dominance on TMS measures of intra-cortical inhibition in right-handed and left-handed individuals. Interestingly, much like in the present study, these authors observed a similar rightward increase in rMTs in their participants regardless of handedness. The reason for this threshold asymmetry, particularly in left-handers, remains difficult to explain, but might be related to observations that left-handers tend to use their less preferred hand (i.e., right hand) more often than right-handers do, especially in tasks requiring fine visuo-motor control [e.g., 34]. In a recent study, Daligadu et al. [35] compared RCs in groups of right- and left-handers and found an asymmetry in excitability that favoured the non-dominant hemisphere over the dominant hemisphere. They reasoned that this asymmetry might represent a difference in motor representations whereby the non-dominant M1 may possess higher excitable elements confined to a smaller area whereas the dominant M1 would possess a larger representation composed of less excitable elements. While the observed threshold asymmetry in our LH group is consistent with this suggestion, we did not find, as reported by Daliglu et al, an asymmetry in RCs between hemispheres, which again highlights the difficulty in drawing any firm conclusion about lateralized differences in corticospinal excitability.

As for the other TMS measures obtained in the activate state, both MEP_facil_ and cSP were found to be of similar magnitude between hemispheres and neither showed evidence of asymmetry in relation to degree of handedness. Consistent with these observations, Priori et al. [36] found no difference between hemispheres in MEP facilitation elicited during tonic contraction in the FDI in both right- and left-handers, although they did report an asymmetry related to handedness for the SP, which was shorter in duration in the dominant hand/hemisphere. We did observe the same trend for shorter SP durations in the dominant hand/hemisphere in our groups, especially in the RH group, but the overall difference was not significant. This discrepancy between our results and those of Priori et al. [36] might be explained by the fact that they used different test intensities to assess SP durations (from 1 to 1.5×MT), whereas our measurements were based on a single test intensity (i.e., 1.2×MT). In fact, our observations are more in line with those of Braune and Fritz [37], who found a high degree of correspondence between hemispheres when measuring SP in small hand muscles in a large sample of healthy adults participants (n = 75). Thus, it seems that cortical circuits mediating inhibition during the SP are not subject to strong laterality effects in relation to handedness.

### Handedness and Hemispheric differences in Transcallosal Inhibition

With regard to interactions between hemispheres, our observations revealed no asymmetries in relation to degrees of handedness in iSP measurements reflecting the onset (LTI) and depth (DTI) of transcallosal inhibition. Using bi-focal paired-pulse stimulation, De Gennaro et al. [12] made similar observations with regard to transcallosal inhibition and handedness, their results showing no differences between hemispheres in both right- and left-handers. The same report did find asymmetries, however, but these concerned only intrahemispheric measures (i.e., rMT and MEP amplitude), which led De Gennaro and colleagues to conclude that handedness was associated with asymmetries in corticospinal excitability but not in transcallosal inhibition. Interestingly, the Bäumer et al. [33] study, which we referred to earlier, reached the opposite conclusion, their findings pointing to a lateralized asymmetry in transcallosal inhibition in right- and left-handers, whereas intrahemispheric measures of excitability were not influenced by handedness. Again, such conflicting results illustrate the difficulties in trying to establish a link between handedness and lateralized differences in corticomotor excitability in TMS studies.

Although the current observations revealed no asymmetry in transcallosal inhibition between hemispheres, interesting differences still emerged when examining variations between groups. Indeed, one of the main findings of this study lies in the observation that participants in the MH group exhibited earlier LTI when compared to participants in either the RH or LH group. In line with this observation, MH participants also tended to show longer DTI, although this trend was not confirmed statistically. Still, both observations concur to suggest faster and deeper hemispheric interactions in the MH group than in the other two groups. Further support for this conclusion comes from the close association between the observed variations in both LTI and DTI measures and variability in LQs; the quadratic nature of the relationship highlighting the differences in the efficiency of transcallosal inhibition between less lateralized as opposed to strongly lateralized participants. In this respect, our observations appear entirely consistent with those of Bernard et al. [15], who observed that less lateralized individuals displayed features at the neurophysiological level that made them distinct from strongly lateralized individuals. For instance, Bernard et al observed that less lateralized individuals showed frequent occurrences of ipsilateral MEPs, a feature that suggests the existence of greater excitatory transcallosal connections in these individuals. Further to this, the increased occurrence of ipsilateral MEPs was associated with faster interhemispheric transfer time, as measured behaviourally with the Poffenberger task. Thus, both the present findings and those of Bernard and colleagues (2011) support the notion that mixed-handedness is associated with faster and more efficient transcallosal communications, as detected with TMS measures. Enhanced transcallosal communications would allow for a greater degree of bi-hemispheric processing for action planning and execution in less lateralized individuals when compared to strongly handed individuals. Such a conclusion is further supported by anatomical evidence showing an inverse correlation between callosal thickness and degrees of handedness; individuals with weaker degrees of lateralization showing larger callosal dimensions in the anterior, mid-body and posterior regions [38].
